# From pen pals to chat rooms: the impact of social media on Middle Eastern Society

**DOI:** 10.1186/s40064-015-1033-4

**Published:** 2015-06-12

**Authors:** Elaine Hatfield, Richard L Rapson

**Affiliations:** University of Hawaii, Honolulu, HI USA

**Keywords:** Social media, Love, Sex, The future

## Abstract

In this article, we will discuss what is known about a surprisingly popular phenomenon in the Middle East—the use of social media to communicate. We will begin with a discussion of what “social media” sites are (sites such as Facebook, Your Middle East, YouTube, Flickr, Muslima.com, chat rooms, and instant messaging) and point out how common they are in the Middle East. Next, we will discuss the reasons why men and women are currently using Internet and social media. Finally, we will discuss what impact social media have had on politics, political dissent, education, and men’s and women’s relationships—and the impact they might be expected to have in future years. Finally, we will focus on the impact of such media on men’s and women’s relationships—including cross-gender friendships, romantic relationships, and sexual relationships.

## Background

Let us begin by defining “social media” and discussing their prevalence in the Middle East^a^. One aim of this paper is to point out how popular such sites have become. We suspect that currently most scholars underrate their popularity and ubiquity in this area of the world. A second goal is to provide some ideas as to the impact of the burgeoning social media on the world of tomorrow in the Middle East. But first: what do we encompass in the term “social media”?

Social media are the online technologies and practices that people use to share content, opinions, insights, experiences, perspectives, and media themselves. They are media for social interaction (Cohen 2011, p. 1).

Today, throughout the world, Internet access is ubiquitous. By 2012, the number of Internet users worldwide had reached 2.27 billion. Although in the Middle East the Web is subject to more widespread regional restrictions than in any other area of the world, its popularity is increasing exponentially. In the 5 years from 2007–2012, it grew from 20 to 77 million, a 294% increase, and it is still growing rapidly (Royal Pingdom 2012). A little more than 50% of the Middle Eastern population can now tune into to the Web. Not surprisingly, the use of the Web and social media is especially common among wealthy, young, college educated Middle Easterners. Today’s social media typically offer several ways for members to connect virtually with one another, including both synchronous (e.g., instant messaging, texting, video chat) and asynchronous (e.g., email) modes of communication.

## Methods

Our first step was to conduct computer searches of the terms: “social media Middle East” and “social media” paired with the names of all of the individual countries listed in endnote 1. We then replaced “social media” with “date and mate matchmaking,” “chat rooms,” “instant messaging,” “speed dating,” and so forth (again paired with names of the individual countries), utilizing the PsycINFO database (American Psychological Association 1967–2010) and MEDLINE (National Library of Medicine 1966–2014) and search engines such as Google, GoogleScholar, Safari, Explorer, Firefox, and Netscape. We also search for the terms “future predictions,” and the like with all the Middle Eastern countries. When all was said and done, we were able to identify a number of papers that assessed people’s attitudes toward computer match making, it’s prevalence, its unique forms in the Middle East, it’s pros and cons, and the impact that it has had and is expected to have on the Middle East. On occasion we wrote to the authors themselves, asking if they had done more work or knew of more work on this topic that had not appeared on our list. Surely more studies exist, but we have been unable to find them. On occasion, when no studies were available for a given country, we were forced to rely on popular magazine and newspaper articles. We followed the same procedure when attempting to unearth the predictions of futurists and forecasters.

### Who uses social media?

Often scholars write as if the Middle East is a single, homogeneous entity. Yet, throughout this part of the world there is great diversity in religion (ranging from Baha’i, Christianity, Judaism, Mandeanism, Shabakism, Unitarian Druze, Yarsan, Yazdånism, Zoroastrianism, and more, to Islam in its many varieties: Middle East 2015). There is diversity in ethnicity, economic status (ranging from $103,000 GDP per capita in Qatar to $2,500 GD in Yemen and the Gaza Strip), in age of Internet users, levels of education, and the like (Middle East 2015; Paige 2014). People speak Arabic, Aramaic, Armenian, Azerbajani, Balochi, Berber, Greek, Hebrew, Kurdish, Persian, Somali, and Turkish (Middle East 2015). About 20 minority languages are also spoken in the Middle East. In a few minutes strolling through the bazaars, one can sometimes brush up against people who would look at home in Biblical to Futuristic times. In modern times the Middle East is a culturally, politically, economically, and religious, and strategically sensitive area. Thus, in this article, although we will draw some general conclusions, in most of our examples we will attempt to specify which of the Middle Eastern groups we are writing about.

Today, in the Middle East, rates of increase in the use of the Internet may be greater than anywhere in the world. Usage ranges from a full 98.6% of the population (in Bahrain), 95.7% (in UAE), and 95% (in Qatar) to a low of 9% (in Iraq) (Internet World Stats 2014; OpenNet Initative 2009). There are marked cultural differences in the use of social media. According to a 2013 survey, there are huge regional differences in the use of Facebook, Twitter, and Google+ (Elmasry et al. 2014). In the Middle East, the relative power of men and women is also very different. There are currently large gender disparities (country by country) in economic participation and opportunity, educational attainment, health and survival, political empowerment, and gender roles between men and women (World Economic Forum 2014). Such disparities naturally affect men’s and women’s interest in and access to the social media.

**Figure 1:**
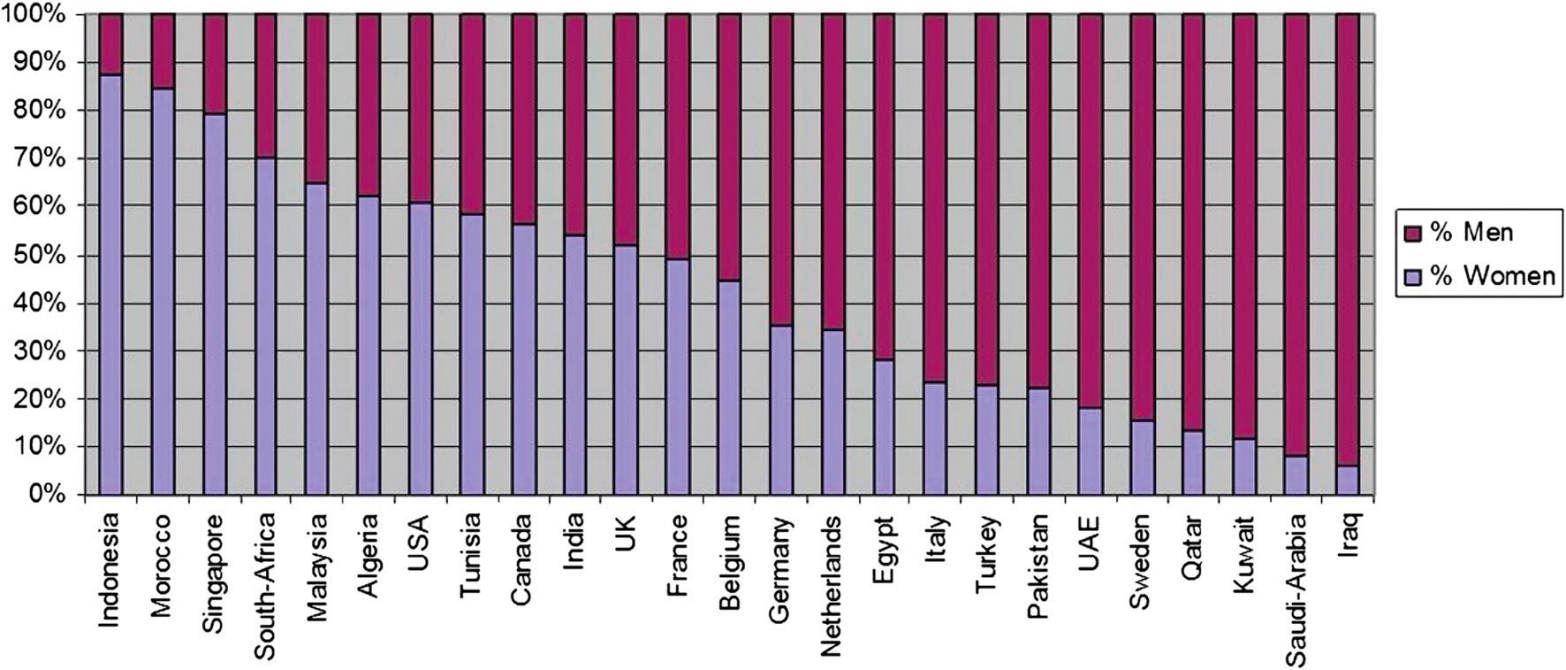
Percentage of men and women subscribing to Muslima.com by country of residence. Courtesy of Diminescu and Renault (2011, p. 693).

Men are the prime users of the Internet and in a most pronounced way, in Middle Eastern dating and chat sites—the topic in which we are most interested in this paper. In fact, in a survey of subscribers to muslima.com, for example, Diminescu and Renault (2011) found that in Middle Eastern countries, virtually all the users were men (see Figure 1). This means that the few women who dare to sign into the new media will have a wide selection of potential mates and people to chat with, while men will have very few alternatives.

Nonetheless, regardless of culture and gender, throughout the Middle East there is a strong current sweeping all young people toward a greater use of the Internet and social media.

### Attitudes toward social media

In the Middle East, parents’ and young people’s attitudes toward social media are complex, to say the least. Most Middle Eastern cultures are far more conservative than are Western cultures. Men and women are sometimes forbidden even to meet before marriage. Certainly they are not expected to have casual chats with people of the opposite sex (Abu-Lughod 1998). Young men and women in more restrictive Middle Eastern countries are aware that they risk severe governmental and parental sanctions should they choose to communicate (and perhaps socialize) with potential romantic or chat partners (Kaya 2009). In Saudi Arabia, for example, there is strict segregation of the sexes. A young woman can get arrested, not to mention getting lashed, for being alone with a man in, say, a restaurant. State-sponsored religious police enforce strict segregation of the sexes (Parssinen 2009). Yet, experiment women do.

In the most conservative countries, paradoxically, online social networks such as khtabh.net are booming. Currently, more than 200 Twitter sites and dozens of other Internet forums offer services for Saudi men and women seeking mates for traditional marriages or simply looking for “the forbidden” chat.

In prosperous Kuwait, most university students are comfortable using the Internet. A full 75% of them are active social media users (Abbas 2001). More than 30% of them (here, more women than men) use it regularly to chat with and even to arrange meetings with members of the opposite sex (Wheeler 2005).

In Cairo, Egypt, in a sample of students at American University, 50% of men and women said they had met at least one member of the opposite sex through the MSN site, a general site owned by Microsoft network, which recently added a free ME personal profile/dating section. (It should be noted that AUC is one of Egypt’s most prestigious and expensive universities.) The MSN site includes an instant messenger section, which enables instant online conversations. Also popular are ICQ (Middle East Chat) and #IRMC, which are instant chat rooms. Only a small sliver of students experimented with specialized dating sites, however—namely cupidjunction and one2onematch (Galal 2003).

In modern-day Israel, most non-traditional Jewish men and women are allowed to meet in public places. There are numerous online dating sites that connect daters and people who simply want to chat with people they might never meet. In Israel, these include MeetIsraeliSingles.com, Jmatch.com, Jdate.com, seeyouinisrael.com, SawyouatSinai.com, and Jwed.com (Bokek-Cohen et al. 2007; Bryant 2013).

Currently, throughout the Middle East, there are several popular Web matching sites and chat rooms (such as Muslims4marriage.com, LoveHabibi.com, Singlemuslim.com, Muslima.com, MuslimLounge.com, and MeetIsraeliSingles.com). The most popular sites for Middle East matching are the US based http://www.zawaj.com (with Web traffic at 2,800,000 page views per month) and http://www.MuslimMatch.com (which has attracted 47,649 members) (Shahine 2004). Another dating and marital matching site is the Egyptian, Arabic-language website El-Nos_El_Tani. When young men and women use social networking sites, they often do so with a uniquely Middle Eastern flair. Elmasry et al. (2014), for example, points out that most women sign on to these sites simply for the chance to chat with men, who they would normally be forbidden to talk to as friends. They do not do so for matchmaking purposes. And it is usually politics and world affairs they talk about, not personal affairs and social life.

### Barriers to the use of social media

As we observed previously, in the various Middle Eastern countries, the “rules” differ by social class, urban/rural status, age and marital status (single versus divorced.) According to Islamic Shariah laws, young Muslims who meet in private places can be charged with *khalwat*, or “close proximity,” which carries a fine and/or a prison sentences of several months. For young women in the Middle East, pressures to preserve “reputation” are especially intense (Galal 2003; Shalhoub-Kevorkian 2003).

Generally speaking, most Middle Easterners who participate in online matching or chat rooms risk sanctions. Consequences may range from social stigma to possible imprisonment. In Baghdad, Layla Ahmad, a retired teacher observed:

We are a conservative society… we don’t accept that our daughters meet boys through the Internet. It’s dangerous, and you can’t observe your children and what they are talking about.

Three months ago, I discovered that my daughter was chatting with somebody online… I took her computer and sold it (Sabah 2006, p. 1)

Of course, as in the West, social media may prove a risky business. Participants must be wary of online predators who disguise their identity or intentions. In a study of Cairo university students, Galal (2003) interviewed men and women, asking them why the avoided matching sites, chat rooms, and meeting people (face-to face) that they had been chatting with online. Women were most concerned about family disapproval and whether they could trust a potential suitor or friend. Men, on the other hand, were most concerned about rejection and concerned that their real-life partners would find out that they had lied to them. As one of our reviewers observed, in this region, issues of privacy and security abound. For instance, when women and men use social media in these countries, they often want to hide their real identity to be safe from family, authorities, or criminals. How possible is this? How much do people worry and how do they try to use social media but hide their true identity from family and friends, or government? This important question is a subject for another paper.

For a detailed discussion of all of the potential difficulties and dangers associated with online matching (in the West), see Finkel et al. (2012) and Sprecher et al. (2008) and (in the Middle East), Galal (2003). Nonetheless, the world is changing, and more Middle Eastern men and women are beginning to use cell phones, social media, visit nightclubs, and access computer matching sites in order to meet potential romantic partners (Peter 2009). Today, many “how to” guides advise Middle Easterners on ways to avoid potential problems. They suggest such things as parental conversations with prospective suitors, chats and instant messaging, telephone calls, chaperoned meetings, and the like (Galal 2003). For a comprehensive list of the problems many Muslim parents, *ulama* (religious leaders), and young people see in use of the Internet, see Galal (2003) and Larsson (2011) .

Despite the potential political, social, and legal problems often associated with Middle Eastern Internet use, the industry continues to flourish. The impact of this technology on social life has arguably been notable in several important ways. By creating venues for men and women to engage in anonymous, one-on-one, communication, social media are altering the ways in which Middle Eastern men and women perceive, understand, and relate to one another (Larrson 2013), thereby changing, particularly, how young people approach relationships and mate selection. What has long been all but unattainable for most young Middle Easterners—direct male–female dialogue—is now readily available with the click of a button. Referring to the influence of Internet dating on Kuwaiti society, Wheeler (2005) explains:

[We] are seeing important signs of experimentation which cannot help but stimulate processes of change over time as young people redefine norms and values for future generations (p. 2).

The advent of social media is clearly changing how the genders communicate with each other in most of the Middle East. In discussing the sweeping changes within the region, de Muth (2011) notes that:

…with thousands of young, single Muslims signing up [with Internet dating/matchmaking sites and chat rooms] every day….there is little chance of putting this particular genie back in the bottle (p. 2).

### The future: what impact might social media have on the Middle East?

People have always had some contact with outside cultures—think of pen pal letters to fellow students in foreign lands. Today, however, as we have seen, pen pals have been replaced by chat rooms—which have an immediacy, power, and ubiquity never before seen. Everyone agrees that this will produce dramatic global changes—but what kinds of change? Will the expansion of the Internet engender wide and deep social changes in the culture and politics of the conservative Middle East? Will it contribute to greater individualism, more freedom, moves toward gender equality, and growing harmony in the long run? No one knows the answers to those questions, but it does not require a huge intellectual stretch to imagine such possibilities. The emerging global culture is based—for better or worse—on those Western values, and perhaps someday the Middle East will be more of a part of that global village (Hatfield and Rapson 2005; Hatfield et al. 2007).

Yale historian Robin Winks once observed that writing history is “like nailing jelly to the wall” (Hatfield 2012). But, he added, “someone must keep trying.” Trying to describe sweeping historical trends and then to predict future trends in even more difficult. But let us, in a playful and modest spirit, make the effort.

On previous occasions—at a NATO conference in 1974 (Hatfield and Walster 1974), and in a century-end issue of *Popular Mechanics* (Hatfield E, Rapson RL: The future, 2000, unpublished), we were asked to make some predictions as to what the world would be like 25–50 years in the future. Our predictions on both occasions possessed the same two flaws that all such efforts do. On one hand, our 1974 predictions vastly underestimated the pace of change (predicting, for example, that by Year 2000, some people might have massive, refrigerator sized IBM computers in their homes) and at the same time we vastly overestimated the pace of change (predicting that by Year 2000, human brain/computer merging would be common). Yet, here are some likely projections:

*General societal changes* A number of theorists have predicted that social media will strengthen global democracy. It will encourage citizenship, genuine communication, and participation in the political process (see The European Commission 2000). Isolation is the major weapon of dictators, such as Kim Jong Un, in North Korea, who attempt to gain power and keep change out. Rahimi (2003), for example, argues that the Internet has already played a critical role in the ongoing struggle for democracy in Iran.

… the internet has opened a new virtual space for political dissent. The paper claims that the Internet is an innovative method for resistance in that it essentially defies control and supervision of speech by authoritarian rule, seeking to undermine resistance (p. 101).

In the Arab world, the Internet is also a vehicle for empowerment for Arab women. Wheeler (2005) interviewed women in Internet cafes in Cairo, Egypt and Amman, Jordan, and concluded:

Areas where Arab women are using the Internet to re-shape their lives, and the lives of others, include health-related concerns; the promotion of civic discourse through open, frank discussions on politics; relationships; and sensitive social issues, both at home and abroad… One of the most important reasons for surfing the web in a café is to meet a partner. This is the reason that “Romance’ is the most popular chat room listed by Arab women…. The Internet remains an important tool for Arab women seeking entertainment, alternative realms for open self-expression, networking possibilities, and someone to love (p. 35).

Some theorists contend that “savy users” (those who are comfortable with social media) will not be colonized by Western values but will adopt a hybrid model—picking and choosing in an informed way among the cultural elements they prefer (Ess 2005). Even today on Facebook, Middle Eastern students in Quatar and Egypt reflect more conservative norms than do American users. Facebook pages in Egypt are more politically oriented, while American pages tend to focus more on social life and personal activities (Elmasry et al. 2014).

Some skeptics, of course, have argued that this scenario of social media as a harbinger of peace, democracy, and a better world is overly optimistic. They point out that ruthless dictators will possess the ability to use their power to censor the Internet and suppress the messages of advocates for freedom (Rolland 2005). Terrorists can (and do) organize via insurgent networks, secret chat rooms, and encrypted message boards, adding to governmental instability (Jakes and Goldman 2013; Weimann 2004). For a discussion of these issues, see Teitelbaum (2002: Saudi Arabia). The Internet is a technology; its uses can be manipulated. The “Arab Spring” revolt did not eventuate in an explosion of democracy in the region. Not yet.

Nonetheless, it is perhaps in the area of male–female friendships and in romantic and sexual relationships that social media is predicted to have the biggest impact.

Thus far, we have discussed the fact that social media are more popular in the Middle East than one might expect. We have discussed who uses the social media, current attitudes toward its use, factors that promote and inhibit its use, and the changes it has brought about in the Middle East. In the final section, we will see what social changes we might expect as social media become an ever-increasing presence in the lives of men and women.

### The future: changes in gender relations, love, and sexual attitudes and behavior

For many years we have asked futurists and our students in Psychology and History classes to attempt to predict what things will be like in the realms of love and sex in the next 25–50 years—based on their extrapolations from current trends. They have identified the following changes as “most probable” (Hatfield 2012). Since no one can in fact predict the future, we urge that these guesses, made a few years ago and offered with the aim of stimulating thought, also be read with skepticism and bemusement. We submit these guesses in hopes of stimulating reflection.

**Cultural and attitudinal changes**

- Acceptance of more definitions of “family.”
- The improved status of women worldwide (Sakr 2014).
- Increasing acceptance of gays, lesbians, and bisexuals.
  Currently homosexuality is a crime in many of the Middle Eastern states, punishable by death in Sudan, Saudi Arabia, Yemen, Qatar, Kuwait, and Iran (Simmons 2014). That may eventually change. Already, sites such as Fatiha.net and Queer Jihad have attracted considerable attention. Irshad Manji has used the site Muslim-Refusenik.com to publicize her profile as a “lesbian feminist Muslim” (Bunt 2004).
- Increasing acceptance of inter-racial and inter-religious relationships.
- Men and women more experienced with love, sex, and intimate relationships

The global village created by worldwide communication, computers, and satellites, information exchange, travel, and trade will most certainly continue to reduce cultural differentiation and augment homogenization. While we can anticipate that the world of the future might combine something of East and West, there can be little doubt that in the short run, in the area of passionate love and sexual desire, the influence of the West on the East will be far greater than the reverse. For some that is an appealing vision. They equate Westernization with freedom, equality, democracy, and higher living standards. For others, that is a nightmare vision, an image of selfishness, rampant greed and materialism—made in the West. Our guess is that people will choose to move toward the Western model, but modified by indigenous traditions.

**Economic/practical**

- Movement towards gender equality
- Both spouses working
- More consensual unions
- More long-distance relationships
- More cyberspace relationships

**Technological**

Cultural and economic changes always occur, but not always in a linear fashion. Technological changes *do* tend to be linear, and are more predictable than other forms of historical change. The best science is usually the latest science; similarly with the best technology. Latest is usually the best and one can imagine what lies down the road. Science fiction is almost always based on predictable linear technological changes. One cannot make similar predictions with any sort of accuracy when it comes to music or literature or relationships or politics or matters of war and peace (see Kurzweil 2006; Toffler 1970, 1980; for a further discussion the this point.). We would expect:

- More love, sex, and relationships on the Web or its more revolutionary descendants.
- Increasing acceptance of cosmetic surgery.
- Sex dolls: Choosing fantasy mates over real men and women
- Humans will fall in love with robots, have sex with robots, and marry robots, and all will be regarded as “normal” expressions of love and sexual desire for other humans (Levy 2007).
- Computer matching
  Currently there are social media sites designed to appeal to various age groups (HookUp.com, SilverSingles.com), political groups (ConservativeMatch.com, LiberalHearts.com), religious groups (CatholicSingles.com, Jdate.com, ChristianCafe.com, HappyBuddhist.com), and sexual orientation (GayWired.com, superEva.com). Chat and dating sites also exist for people who possess mental and physical disabilities, unusual sexual preferences, and so forth. Even people who wish to find dates for themselves and their favorite pets can sign on to a site (DateMyPet.com)! At the time this chapter was written, there may be almost 1,000 dating websites servicing the US. In future years such a plethora of sites will be available to all.
- Increased availability of pornography and technological sex
- Cures for AIDS, STIs, and sexual dysfunction
- Advances in reproductive technology—including birth control and abortion technology. One will be able to design boutique (and “test-tube”) babies.
- People will live longer. Much longer and healthier.
  The vision of people with so many artificial parts that man and machine will merge may become a reality. See Kurzweil’s (2006) speculations about the singularity, which he predicts will arrive around 2045.
- Above all, the global norm will be *change*—probably very rapid change. Many people will experience what the futurist Toffler (1970) called “future shock.”

Since love and sex have historically been such fraught issues and change in these areas has not always moved in a steadily linear fashion—only science and technology do that (see Kurzweil 2006; Toffler 1970, 1984, for a further discussion the this point). It may take 100 years rather than 25 for all the above changes to take root, but for better or worse, take root they likely shall

## Conclusions

In an invited address at the American Psychological Association, Hazel Markus lamented the fact that most psychological theories and studies were conceived by Americans, tested with American (and white) college students, and published in American psychology journals. “Even the rats were white,” she joked. In this study, we attempted to expand the horizons of social psychologists. If questioned, perhaps most Americans would be uncertain if social media and chat-rooms even existed in the Middle East. They would certainly not know how popular they are and how commonly they are used. In this paper, we provided that information; the sites are surprisingly popular. We also speculated as to the impact that the popular usage of such sites may be expected to have on Middle Eastern society.

## Endnote

^a^In this paper, we will include Afghanistan, Algeria, Egypt, Iran, Iraq, Israel, Jordan, Libya, Morocco, Pakistan, Palestine, Saudi Arabia, Syria, Tunisia, Turkey, and the UAE, in our definition of “Middle East”.
